# Medication Prescription Policy for US Veterans With Metastatic Castration-Resistant Prostate Cancer: Causal Machine Learning Approach

**DOI:** 10.2196/59480

**Published:** 2024-11-19

**Authors:** Deepika Gopukumar, Nirup Menon, Martin W Schoen

**Affiliations:** 1 Richard A Chaifetz School of Business Saint Louis University St. Louis, MO United States; 2 School of Medicine Saint Louis University St. Louis, MO United States; 3 Costello College of Business George Mason University Fairfax, VA United States; 4 St Louis Veteran Affairs Medical Center St. Louis, MO United States

**Keywords:** prostate cancer, metastatic castration resistant prostate cancer, causal survival forest, machine learning, heterogeneity, prescription policy tree, oncology, pharmacology

## Abstract

**Background:**

Prostate cancer is the second leading cause of death among American men. If detected and treated at an early stage, prostate cancer is often curable. However, an advanced stage such as metastatic castration-resistant prostate cancer (mCRPC) has a high risk of mortality. Multiple treatment options exist, the most common included docetaxel, abiraterone, and enzalutamide. Docetaxel is a cytotoxic chemotherapy, whereas abiraterone and enzalutamide are androgen receptor pathway inhibitors (ARPI). ARPIs are preferred over docetaxel due to lower toxicity. No study has used machine learning with patients’ demographics, test results, and comorbidities to identify heterogeneous treatment rules that might improve the survival duration of patients with mCRPC.

**Objective:**

This study aimed to measure patient-level heterogeneity in the association of medication prescribed with overall survival duration (in the form of follow-up days) and arrive at a set of medication prescription rules using patient demographics, test results, and comorbidities.

**Methods:**

We excluded patients with mCRPC who were on docetaxel, cabaxitaxel, mitoxantrone, and sipuleucel-T either before or after the prescription of an ARPI. We included only the African American and white populations. In total, 2886 identified veterans treated for mCRPC who were prescribed either abiraterone or enzalutamide as the first line of treatment from 2014 to 2017, with follow-up until 2020, were analyzed. We used causal survival forests for analysis. The unit level of analysis was the patient. The primary outcome of this study was follow-up days indicating survival duration while on the first-line medication. After estimating the treatment effect, a prescription policy tree was constructed.

**Results:**

For 2886 veterans, enzalutamide is associated with an average of 59.94 (95% CI 35.60-84.28) more days of survival than abiraterone. The increase in overall survival duration for the 2 drugs varied across patient demographics, test results, and comorbidities. Two data-driven subgroups of patients were identified by ranking them on their augmented inverse-propensity weighted (AIPW) scores. The average AIPW scores for the 2 subgroups were 19.36 (95% CI –16.93 to 55.65) and 100.68 (95% CI 62.46-138.89). Based on visualization and *t* test, the AIPW score for low and high subgroups was significant (*P*=.003), thereby supporting heterogeneity. The analysis resulted in a set of prescription rules for the 2 ARPIs based on a few covariates available to the physicians at the time of prescription.

**Conclusions:**

This study of 2886 veterans showed evidence of heterogeneity and that survival days may be improved for certain patients with mCRPC based on the medication prescribed. Findings suggest that prescription rules based on the patient characteristics, laboratory test results, and comorbidities available to the physician at the time of prescription could improve survival by providing personalized treatment decisions.

## Introduction

Prostate cancer is the second leading cause of cancer death among men in the United States and the most common cancer affecting men of African descent [1]. While early-stage prostate cancer is curable, around 10% to 50% of cases progress to metastatic castrate-resistant prostate cancer (mCRPC) within 3 years of diagnosis, which is fatal [2]. Multiple therapies exist for treating mCRPC, including androgen-receptor pathway inhibitors (ARPIs) and cytotoxic chemotherapy, such as docetaxel. ARPIs, such as enzalutamide or abiraterone, target the androgen receptor signaling pathways and are administered orally [3]. Enzalutamide is administered at 160 mg orally once daily [4]. In comparison, abiraterone acetate is administered while fasting at a dose of 1000 mg orally with coadministration of the steroid prednisone [3]. Recent prescription trends show that ARPIs are preferred for their tolerable safety profiles and improving survival [5,6]. Prescribing ARPIs instead of docetaxel might also be beneficial for African American men, though the precise mechanisms are yet known [7].

Abiraterone and enzalutamide have different mechanisms of action. Abiraterone requires coadministration of prednisone because it inhibits androgen biosynthesis, leading to mineralocorticoid excess. In contrast, enzalutamide inhibits the androgen receptor by blocking hormone signaling and does not require steroid coadministration. These differences result in varying patient outcomes and survival based on patient demographics, test results, and comorbidities [8]. Clinical trials often do not encompass the full spectrum of patient comorbidities [9]. In the absence of clear clinical evidence favoring one ARPI over the other, retrospective observational data and data analytics techniques can help determine the most suitable drug for individual patients [10].

The methods used to study outcomes using ARPIs included associative and predictive modeling. Association studies identified a few comorbidities, such as cardiovascular diseases and diabetes, as significant predictors of survival in patients with mCRPC and comorbid diseases [10]. In addition, studies of hospitalizations during ARPI treatment showed an increased risk of heart failure, atrial fibrillation, and acute kidney injury with abiraterone [11]. Enzalutamide was found to be better than abiraterone for survival as well [10,12].

Machine learning predictive models effectively predicted survival outcomes and time to treatment discontinuation using tree-based approaches while incorporating laboratory test results such as hemoglobin and albumin and comorbidities such as hypertension and diabetes [13-15]. Multiomic features combined with treatment lines predicted the response types “good, poor, and ambiguous” following ARPI treatment [16]. However, these studies did not identify patient-specific differences in outcomes (heterogeneous treatment effects in subgroups of patients), address selection bias in treatment, or prescribe specific ARPIs based on patient demographics, laboratory test results, and comorbidities.

The main objective of this study was to evaluate patient-level heterogeneity in the association between prescribed medication and overall survival duration in the form of follow-up days and subsequently to identify a set of prescription rules based on patient-specific factors. We used a causal survival forest to measure heterogeneous treatment effects among patients with mCRPC, focusing on the survival duration. Based on these findings, we developed a set of treatment rules, or a policy tree, to prescribe abiraterone and enzalutamide tailored to individual patient factors such as demographics, test results, and comorbidities.

## Methods

### Dataset and Its Description

The data used for analysis were from the United States Department of Veterans Affairs (VA) centers (compassing VA hospitals and clinics) stored in the Corporate Data Warehouse. A total of 3675 veterans from Corporate Data Warehouse were identified as patients with mCRPC, excluding those with missing values and including only the African American and White populations. We excluded patients with mCRPC who received docetaxel, cabazitaxel, mitoxantrone, or sipuleucel-T before or after starting abiraterone or enzalutamide. This exclusion resulted in a dataset of 2890 veterans who began treatment with abiraterone or enzalutamide between 2014 and 2017. In addition, 4 patients were excluded because they switched to different second-line treatments on the same day. Thus, the final analysis included 2886 patients. The study follow-up concluded in 2020.

#### Patient Demographics, Test Results, and Comorbidities

In total, 20 covariates’ age, creatinine clearance test result category, albumin result category, bilirubin result category, hemoglobin result category, race, prostate-specific antigen (PSA) test, BMI category, diabetes, hypertension, kidney disease, osteoporosis, fall, fatigue, abnormal gait, peripheral neuropathy, Parkinson’s disease, vision, orchiectomy procedure, and cardiovascular diseases (CVDs) were used. The laboratory values were calculated before treatment and were closest to the start of the first-line treatment. Covariates, such as CVD and diabetes, have been previously used to study the survival of veterans with mCRPC in univariate studies [10]. Other covariates such as abnormal gait, peripheral neuropathy (a common side effect of cancer treatment), and vision are included to capture existing comorbidities better. CVD was calculated based on Charlson and Elixhauser indices for myocardial infarction, heart failure, cardiac arrhythmia, valvular disease, complicated hypertension, peripheral vascular disease, and cerebrovascular disease based on *ICD* (*International Classification of Diseases*) codes [10]. Other comorbidity-related covariates were based on the standard VA-Frailty health deficits based on diagnosis codes. We used proxies for comorbidities not available in our dataset but have been studied in the past, such as alkaline phosphate level. For example, kidney disease is a proxy for alkaline phosphate levels [17].

#### Outcome

The primary outcome of our study was overall survival duration, measured in follow-up days from the initiation of first-line treatment with an ARPI (abiraterone or enzalutamide). For a subset of patients with mCRPC (1163 out of 2886) who received the other ARPI drug as second-line treatment following the first-line therapy, we considered follow-up days to the initiation of the second-line treatment as survival duration. This approach assumed survival up to that point without accounting for follow-up days post-second-line treatment, aiming to mitigate treatment switching bias between therapies [18]. In addition, our data were limited to laboratory results and comorbidities recorded on the day of first-line treatment initiation.

### Statistical Analysis

We used causal survival forests with 20 covariates to estimate heterogeneous treatment effects in follow-up days [19]. Causal forests and causal survival forests are similar to random forests and random survival forests, respectively, with the primary difference being their data splitting criteria [20]. In a random survival forest, data splitting minimizes differences in prediction errors in follow-up days within each group, considering censoring. In contrast, a causal survival forest splits data to maximize heterogeneity, that is, the difference in the estimated follow-up days between the treated and untreated groups, accounting for censoring. The advantage of using a causal survival forest lies in its robustness to censoring, unlike a causal forest [19]. In addition, compared with a random survival forest, a causal survival forest offers a more comprehensive assessment of heterogeneity. It can outperform linear regression models by measuring heterogeneous treatment effects conditional on a nonlinear function of many covariates, accounting for higher-order interactions [21].

Each observation in the dataset corresponds to a single patient with mCRPC and includes the patient’s covariate data, follow-up days (the outcome), treatment assignment (1 for enzalutamide, 0 for abiraterone), and death (event). Survival duration was analyzed based on either abiraterone or enzalutamide treatment without an untreated control group. Consequently, the dataset does not allow for the determination of the individual treatment effects of each drug.

As this is a retrospective observational study, we must account for the nonrandomization of treatment assignment and use the augmented inverse-propensity weighting (AIPW) estimator, a doubly robust method. To do this, the true treatment assignment was fitted as a function of the observed covariates. The predicted value from this model provides a propensity score, that is, an estimate of the probability of treatment assignment conditioned on a set of covariates for each patient. Then, 2 models that estimate the outcome (follow-up) were fitted, one using enzalutamide and the other using abiraterone. Each outcome was then weighted by the estimated propensity score, which yields the weighted average of the two outcome models [22].

We used a causal survival forest for our analysis [19]. To ensure that the assumptions of finite horizon, ignorability, overlap, ignorable censoring, and positivity are satisfied so that the treatment effect of the drugs is identified when using causal survival forests, we made the following analytical choices. The parameter horizon, referring to restricted mean survival time, was set to a threshold so that the estimated censoring probabilities are not below 0.2, satisfying the finite horizon and positivity assumption. We started with a horizon of 2000 days (approximately close to the maximum follow-up days) and decremented by 100 to 1000.

For ignorability, we applied balanced diagnostics by checking the weighted absolute standardized mean difference (ASMD) of variables between patients treated with enzalutamide and patients treated with abiraterone. A number close to 0 indicates that the propensity scores are well-calibrated. We examined the propensity score distribution for both groups to test for overlap and checked if they clustered at 0 or 1.

The honesty fraction was set to 0.7, meaning 70% of the subsample was used for splitting and 30% for populating the leaf nodes. However, for ranking the observations based on covariates and coming up with subgroups, the model must not be fit using the observations being compared. We used 10-fold cross-fitting for this reason. The conditional average treatment effects (CATE) models were fit for 9 folds, and the “unseen” observations in the left-out fold were ranked based on their predictions and split along the median into 2 groups. The same process was repeated 10 times, with each fold serving as a left-out fold once.

We set the number of trees to 15,000. We generated the policy tree after calculating the AIPW scores. A policy tree is a set of treatment rules, that is, rule-based policies, in the form of a decision tree that physicians can use to prescribe abiraterone and enzalutamide. The policy tree was generated using 70% of the training data, and the policy’s value was determined using the 30% test data. The policy value is defined as the average difference in follow-up days obtained if the patients with mCRPC are administered enzalutamide or abiraterone. R (version 4.4.1; The R Foundation) with packages *grf* and *policytree* were used for analyses.

## Results

We used a causal survival forest to estimate the heterogeneity in the treatment effect on survival for the 20 covariates (refer to Table 1 for the descriptive statistics and description for all patients including based on enzalutamide and abiraterone).

**Table 1:** Descriptive statistics of metastatic castration-resistant prostate cancer US veterans with androgen receptor pathway inhibitors treatment initiated 2014-2017 (Total sample N=2886; abiraterone n=1649; enzalutamide n=1237).

Predictor and categories			All (entire sample)		Abiraterone (Treatment=0)		Enzalutamide (Treatment=1)
**Race, n (%)**							
	Black	691 (23.94)		380 (23.04)		311 (25.14)	
	White	2195 (76.06)		1269 (76.96)		926 (74.86)	
**Age, years**							
	Minimum-maximum	51-90		51-90		53-90	
	Median (IQR)	78 (69-84)		78 (69-84)		78 (70-84)	
	Mean (SD)	77 (9)		77 (9)		77 (9)	
**PSA^a^ test result**							
	Minimum-maximum	0-7289		0-7289		0-3846	
	Median (IQR)	32 (10-100)		33 (10-105)		31 (9-90)	
	Mean (SD)	138 (399)		147 (449)		127 (322)	
**Creatinine clearance category, n (%)**							
	≥30	2748 (95.22)		1561 (94.66)		1187 (95.96)	
	<30	138 (4.78)		88 (5.34)		50 (4.04)	
**Albumin category, n (%)**							
	≥3	2723 (94.35)		1560 (94.6)		1163 (94.02)	
	<3	163 (5.65)		89 (5.4)		74 (5.98)	
**Bilirubin category, n (%)**							
	<2	2872 (99.51)		1643 (99.64)		1229 (99.35)	
	≥2	14 (0.49)		6 (0.36)		8 (0.65)	
**Hemoglobin category, n (%)**							
	≥10	2507 (86.87)		1433 (86.9)		1074 (86.82)	
	<10	379 (13.13)		216 (13.1)		163 (13.18)	
**BMI category, n (%)**							
	<18.5	70 (2.43)		38 (2.3)		32 (2.59)	
	18.5-24.9	791(27.41)		461 (27.96)		330 (26.68)	
	25-29.9	1055 (36.56)		611 (37.05)		444 (35.89)	
	≥30	970 (33.61)		539 (32.69)		431 (34.84)	
**Kidney disease, n (%)**							
	No	1619 (56.10)		962 (58.34)		657 (53.11)	
	Yes	1267 (43.9)		687 (41.66)		580 (46.89)	
**Osteoporosis, n (%)**							
	No	2618 (90.71)		1491 (90.42)		1127 (91.11)	
	Yes	268 (9.29)		158 (9.58)		110 (8.89)	
**Fall, n (%)**							
	No	2750 (95.29)		1570 (95.21)		1180 (95.39)	
	Yes	136 (4.71)		79 (4.47)		57 (4.61)	
**Fatigue, n (%)**							
	No	2422 (83.92)		1383 (83.87)		1039 (83.99)	
	Yes	464 (16.08)		266 (16.13)		198 (16.01)	
**Abnormal gait, n (%)**							
	No	2345 (81.25)		1346 (81.63)		999 (80.76)	
	Yes	541 (18.75)		303 (18.37)		238 (19.24)	
**Parkinson disease, n (%)**							
	No	2834 (98.2)		1623 (98.42)		1211 (97.9)	
	Yes	52 (1.8)		26 (1.58)		26 (2.1)	
**Peripheral neuropathy, n (%)**							
	No	2682 (92.93)		1540 (93.39)		1142 (92.32)	
	Yes	204 (7.07)		109 (6.61)		95 (7.68)	
**Vision comorbidity, n (%)**							
	No	2127 (73.7)		1239 (75.14)		888 (71.79)	
	Yes	759 (26.3)		410 (24.86)		349 (28.21)	
**Orchiectomy, n (%)**							
	No	2881 (99.83)		1648 (99.94)		1233 (99.68)	
	Yes	5 (0.17)		1 (0.06)		4 (0.32)	
**Cardiovascular diseases, n (%)**							
	No	963 (33.37)		568 (34.45)		395 (31.93)	
	Yes	1923 (66.63)		1081 (65.55)		842 (68.07)	
**Hypertension, n (%)**							
	No	532 (18.43)		316 (19.16)		216 (17.46)	
	Yes	2354 (81.57)		1333 (80.84)		1021 (82.54)	
**Diabetes, n (%)**							
	No	1712 (59.32)		1038 (62.95)		674 (54.49)	
	Yes	1174 (40.68)		611 (37.05)		563 (45.51)	

^a^PSA: prostate-specific antigen.

**Figure 1:**
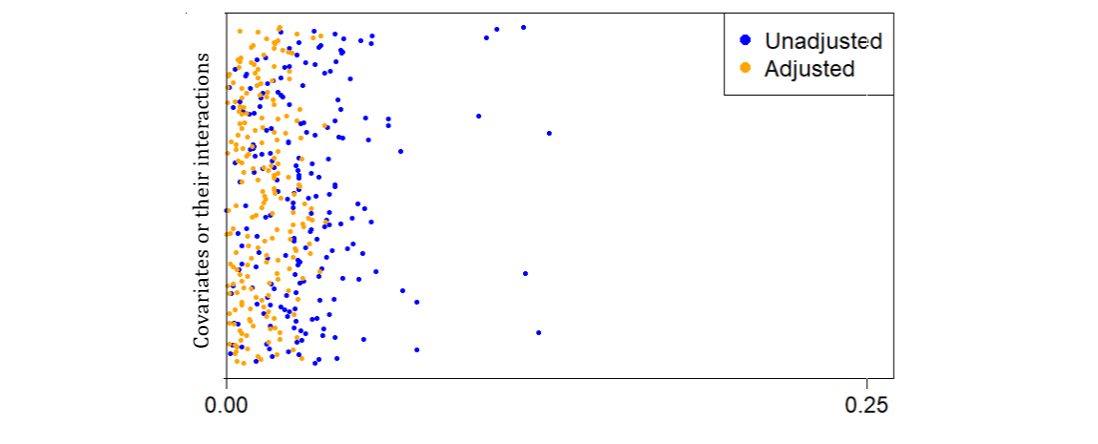
Absolute standardized mean differences of covariates and their interactions between abiraterone and enzalutamide-treated groups. The covariates closeness to zero after adjusting for selection bias (the orange dots) indicates the balance between the 2 groups.

**Figure 2:**
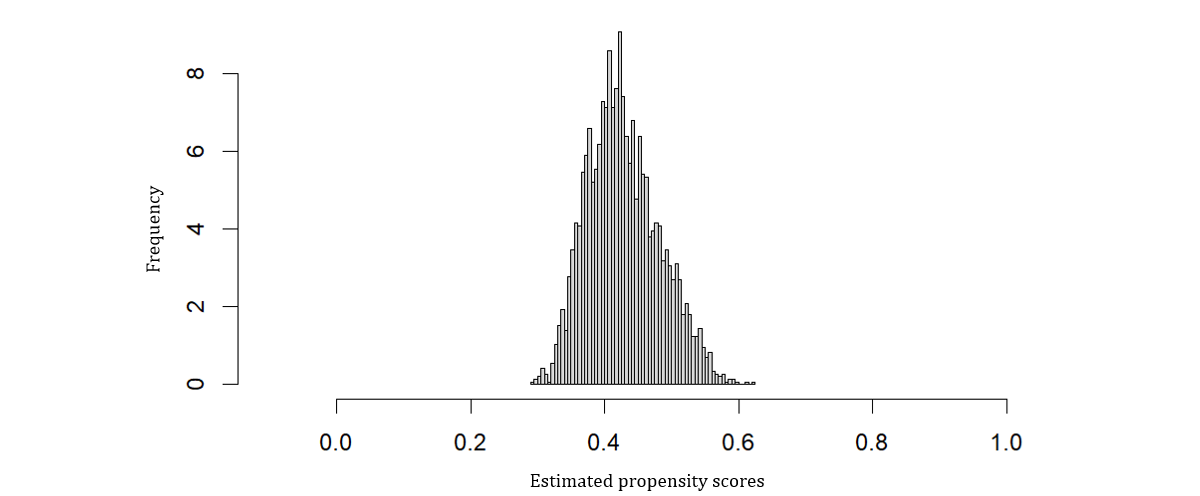
Estimated propensity scores from causal survival forest for metastatic castration-resistant prostate cancer US veterans with androgen receptor pathway inhibitors treatment initiated 2014-2017. The scores do not cluster at 0 or 1, indicating overlap.

We found the horizon value of 1000 to be the most suitable, with the censoring probability estimates not going below 0.2, satisfying the positivity assumption. The weighted ASMD of covariates, along with their interactions, for the abiraterone and enzalutamide-treated groups is close to zero, satisfying the balance between the 2 groups (Figure 1). Thus, the propensity score estimates from causal survival forests were well-calibrated. The plot of the estimated propensity scores showed that the scores did not cluster at zero or one and were unimodal, exhibiting overlap (Figure 2).

**Figure 3:**
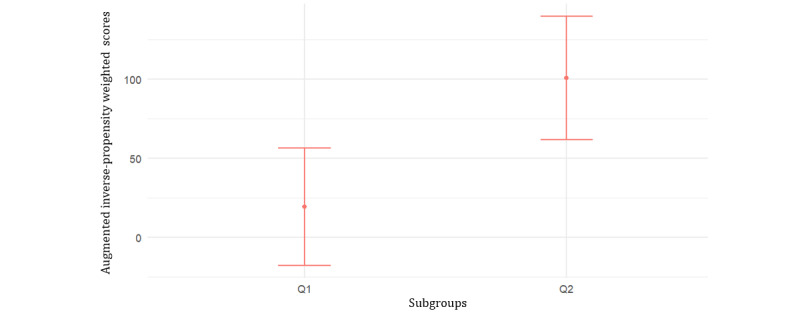
Augmented inverse-propensity weighted scores (mean and 95% CI) for the 2 subgroups above and below the median for all metastatic castration-resistant prostate cancer US veterans with androgen receptor pathway inhibitors treatment initiated 2014-2017.

For the 2886 veterans, enzalutamide is associated with an average of 59.94 (95% CI 35.6-84.28) more days of survival than abiraterone. The CATE scores computed from the causal survival forest were used to rank and split the observations into 2 subgroups, above and below the median [23]. The average AIPW scores for the 2 subgroups were 19.36 (95% CI -16.93 to 55.65) and 100.68 (95% CI 62.46-138.89). Based on visualization (Figure 3) and *t* test, the difference between the AIPW scores for low and high subgroups was significant (*P*=.003), thereby supporting heterogeneity.

**Figure 4:**
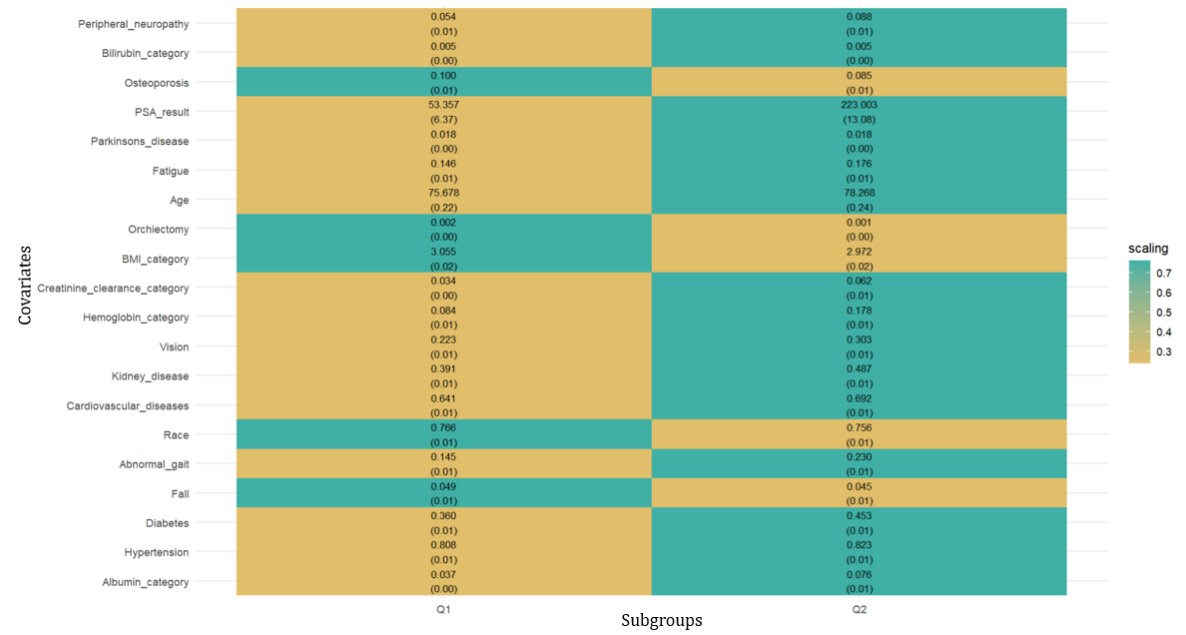
Average covariate values for the 2 subgroups above and below the median augmented inverse-propensity weighted score for all metastatic castration-resistant prostate cancer US veterans with androgen receptor pathway inhibitors treatment initiated 2014-2017.

The heatmap in Figure 4 shows the average value of each covariate within each subgroup (refer to Table 2 for descriptive statistics for the 2 subgroups). The subgroups are ordered from lowest to highest difference in treatment effects; Q2, the second subgroup, has a higher treatment effect than Q1. The color in the heatmap of each covariate is the normalized distance of the average of the covariate in the subgroup from the average of the covariate in the full data. The covariates of the heatmap are arranged in descending order of variation, comparing the variance of the covariate in a subgroup with its variance in the sample. The top 5 covariates contributing to the highest variation were peripheral neuropathy, bilirubin category, osteoporosis, PSA test result, and Parkinson’s disease.

**Table 2:** Descriptive statistics for the subgroup of US veterans with metastatic castration-resistant prostate cancer below and above the median conditional average treatment effects (Total Sample N=2886).

Predictor and categories		Below median CATE^a^ (Ranking=1)	Above median CATE^a^ (Ranking=2)
**Race, n (%)**			
	Black	339 (23.44%)	352 (24.44%)
	White	1107 (76.56%)	1088 (75.56%)
**Age**			
	Minimum-maximum	51-90	51-90
	Median (IQR)	76 (69-83)	80 (71-86)
	Mean (SD)	76 (8)	78 (9)
**PSA^b^ test result**			
	Minimum-maximum	0-7289	0-6240
	Median (IQR)	17 (8-38)	89 (21-203)
	Mean (SD)	53 (242)	223 (496)
**Creatinine clearance category, n (%)**			
	≥30	1397 (96.61)	1351 (93.82)
	<30	49 (3.34)	89 (6.18)
**Albumin category, n (%)**			
	≥3	1392 (96.27)	1331 (92.43)
	<3	54 (3.73)	109 (7.57)
**Bilirubin category, n (%)**			
	<2	1439 (99.52)	1433 (99.51)
	≥2	7 (0.48)	7 (0.49)
**Hemoglobin category, n (%)**			
	≥10	1324 (91.56)	1183 (82.15)
	<10)	122 (8.44)	257 (17.85)
**BMI category, n (%)**			
	<18.5	36 (2.49)	34 (2.36)
	18.5-24.9	374 (25.86)	417 (28.96)
	25-29.9	510 (35.27)	545 (37.85)
	≥30	526 (36.38)	444 (30.83)
**Kidney disease, n (%)**			
	No	881 (60.93)	738 (51.25)
	Yes	565 (39.07)	702 (48.75)
**Osteoporosis, n (%)**			
	No	1301 (89.97)	1317 (91.46)
	Yes	145 (10.03)	123 (8.54)
**Fall, n (%)**			
	No	1375 (95.09)	1375 (95.49)
	Yes	71 (4.91)	65 (4.51)
**Fatigue, n (%)**			
	No	1235 (85.41)	1187 (82.43)
	Yes	211 (14.59)	253 (17.57)
**Abnormal gait, n (%)**			
	No	1236 (85.48)	1109 (77.01)
	Yes	210 (14.52)	331 (22.99)
**Parkinson disease, n (%)**			
	No	1420 (98.20)	1414 (98.19)
	Yes	26 (1.80)	26 (1.81)
**Peripheral neuropathy, n (%)**			
	No	1368 (94.61)	1314 (91.25)
	Yes	78 (5.39)	126 (8.75)
**Vision comorbidity, n (%)**			
	No	1124 (77.73)	1003 (69.65)
	Yes	322 (22.27)	126 (8.75)
**Orchiectomy, n (%)**			
	No	1443 (99.79)	1438 (99.86)
	Yes	3 (0.21)	2 (0.14)
**Cardiovascular diseases, n (%)**			
	No	519 (35.89)	444 (30.83)
	Yes	927 (64.11)	996 (69.17)
**Hypertension, n (%)**			
	No	277 (19.16)	255 (17.71)
	Yes	1169 (80.84)	1185 (82.29)
**Diabetes, n (%)**			
	No	925 (63.97)	787 (54.65)
	Yes	521 (36.03)	653 (45.35)

^a^CATE: conditional average treatment effects.

^b^PSA: prostate-specific antigen.

**Figure 5:**
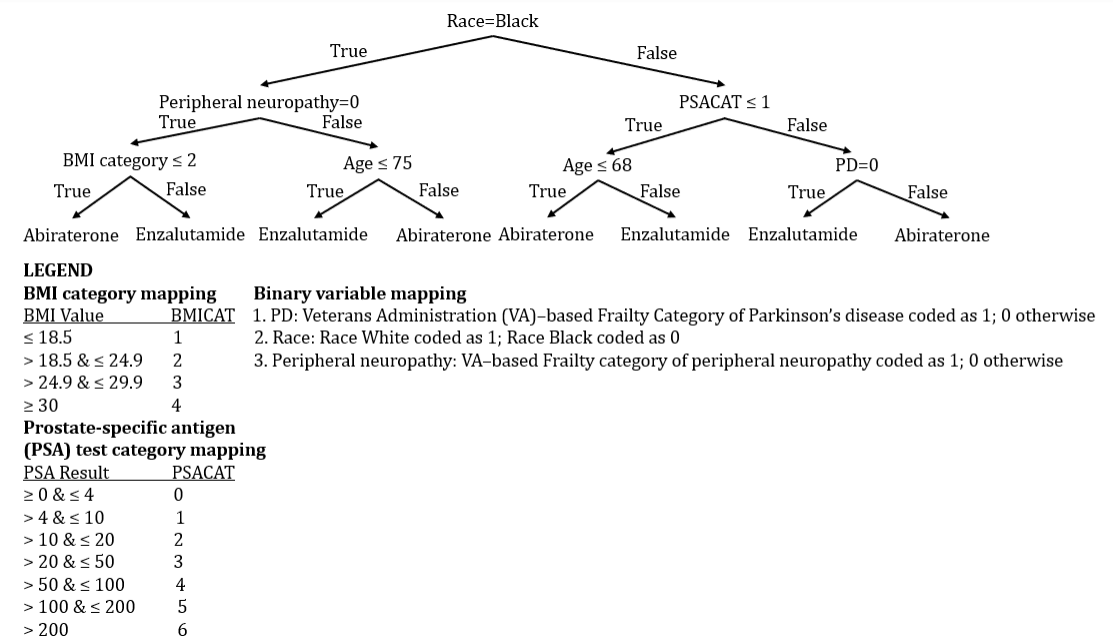
Androgen receptor pathway inhibitors prescription policy tree estimated for US veterans with metastatic castration-resistant prostate cancer.

We estimated the AIPW scores for treatment effects, in terms of follow-up days, for each individual if abiraterone and enzalutamide were administered. Next, we generated prescription policy trees for various depth levels. We used 70% of the data for training to generate the prescription policy tree. To arrive at the value (survival duration) that the policy bestows on a patient, we used the estimated AIPW scores for an individual if administered either abiraterone or enzalutamide based on the predictions from the prescription policy tree. We found significance in the prescription policy tree with a depth of 3. We used a minimum node size of 4. For depth 3 (Figure 5), the average number of follow-up days obtained by administering enzalutamide and abiraterone based on the generated prescription policy tree was 56.38 (95% CI 8.89 to 103.87).

## Discussion

### Principal Findings and Comparison With Previous Work

In this study, we applied a causal survival forest to assess the heterogeneous treatment effects of the mCRPC medications enzalutamide and abiraterone on overall survival. We found that, on average, for 2886 veterans, 59.94 (95% CI 35.60-84.28) more days of survival than abiraterone. The value of our policy is 56.38 (95% CI 8.89-103.87), which is not statistically different from 59.94. This means that the simple, transparent treatment rules using the policy tree method to prescribe abiraterone and enzalutamide can achieve a policy value similar to the conditional average treatment effect. Schoen et al [10] used an adjusted hazard model and found that age, BMI, and PSA level were positively associated with mortality. Our policy tree elicited the decision checks for age, PSA level, and BMI to make prescription decisions on which of the 2 medications is likely to prolong survival.

Interestingly, even though in univariate analyses in previous studies, the median treatment duration was longer in patients with hypertension or diabetes, the policy tree did not choose both hypertension and diabetes and did not contribute to the highest variation between the 2 subgroups. In previous work, increased hemoglobin A_1c_, a marker of diabetes severity, was associated with more prolonged survival in veterans prescribed enzalutamide compared with abiraterone [24]. Instead, our policy tree chose peripheral neuropathy. However, peripheral neuropathy is a complication of diabetes (in addition to being a predictor of severe diabetes, it could be caused by factors such as injury and exposure to toxins and is also a common side effect of cancer treatment) [7,25]. Vision loss can be a complication of diabetes and is associated with increased hospitalizations [26], impaired cognition [27], and higher mortality [28]. To delay diabetic neuropathy, apart from good glycemic control, it has also been found beneficial to control blood pressure [29]. All these could have contributed to choosing peripheral neuropathy instead of diabetes and hypertension by our policy tree.

In addition, our policy tree incorporated osteoporosis, which was also one of the top covariates contributing to the variation between the 2 subgroups. Previous studies show an increased risk of falls and fractures among patients with mCRPC taking ARPIs [30,31]. Osteoporosis and abnormal gait may identify impaired functional status or increased frailty, which is associated with survival outcomes in mCRPC [9]. Osteoporosis is a hidden nonmotor symptom of Parkinsons’ disease, which contributed to variation in subgroups and was chosen by the policy tree [32]. Bilirubin levels could contribute to osteoporosis, which is also shown as one of the top variations among the two subgroups [33]. Our policy tree chose race, and existing studies have demonstrated that abiraterone has improved survival when given as the first line of treatment for the African American population [34]. Together, these results show that our policy tree could elicit a variety of comorbidities and suggest therapy.

In addition to identifying the covariates, selecting treatments for patients with mCPRC using the generated policy tree considering their demographics, laboratory measures, and comorbidities may help mitigate complications and prolong overall survival. As no generic version of enzalutamide is available in the US, the drug remains expensive. Therefore, from a cost perspective, it is prudent to administer enzalutamide only to patients most likely to benefit from it in terms of survival duration.

### Limitations and Future Studies

One of the limitations of this study is that the data are only for US veterans, so that the generalization of the prescription rules to the other populations is not advised. Another limitation is that we excluded patients with mCRPC who had docetaxel, cabaxitaxel, mitoxantrone, and sipuleucel-T before or after prescribing abiraterone or enzalutamide. This was performed to identify patients with higher comorbid disease burdens with fewer therapeutic options. However, other treatments are prescribed for patients with mCRPC. A third limitation of this study was the lack of data on time-varying covariates. Even though causal survival forest is robust to censoring, it will be interesting to include time-varying covariates in the future and to check for differences in the generated policy tree. The fourth limitation of this study was that we used only 70% of the training data to generate a policy tree, because the causal survival forest used cross-fitting and honesty approaches. Furthermore, we estimated the overall benefit of the policy tree (ie, the average difference in the follow-up days obtained if the patients with mCRPC are administered enzalutamide or abiraterone by adhering to the policy tree) in this study without considering the benefits to subgroups of patients based on race or other demographics. Future research should estimate the benefit of such policy trees to see if they are equally beneficial to various subgroups of demographics. Similarly, this study used only mCRPC patient data. Future studies should develop treatment selection for earlier stages of prostate cancer, such as the metastatic hormone-sensitive stage.

### Conclusions

We used a machine learning-based survival approach, that is, causal survival forest model, to estimate the variations in the survival of patients with mCRPC who were administered either enzalutamide or abiraterone. Our estimation revealed that patients with mCRPC who were administered enzalutamide had longer survival than patients who were administered abiraterone. We were able to use a data-driven approach to identify heterogeneity and subgroups of patients. We then created policy trees to aid physicians administer personalized treatment, that is, abiraterone versus enzalutamide, based on patient characteristics, including demographics, laboratory values, and comorbidities, to improve survival duration.

## Abbreviations

AIPW: augmented inverse-propensity weighting
ARPI: androgen-receptor pathway inhibitor
ASMD: absolute standardized mean difference
CATE: conditional average treatment effects
CVD: cardiovascular diseases
ICD: International Classification of Diseases
mCRPC: metastatic castrate-resistant prostate cancer
PSA: prostate-specific antigen
VA: Veterans Affairs

## Data Availability

Scientific community members can request the dataset by e-mailing MWS at martin.schoen@va.gov. It will be shared upon request and execution of an agreement of use. The investigator should state the reason for requesting the data and analysis plans.
